# Supplementary material for: Anaerobic consortia of fungi and sulfate reducing bacteria in deep granite fractures
Source: Nat Commun. 2017 Jul 4;8:55. doi: 10.1038/s41467-017-00094-6 (PMC5496868; doi:10.1038/s41467-017-00094-6)
Supplement: Supplementary file 1 — Supplementary Information [file 41467_2017_94_MOESM1_ESM.pdf]

File name: Supplementary Information
Description: Supplementary Figures, Supplementary Tables and Supplementary References

File name: Peer Review File
Description:

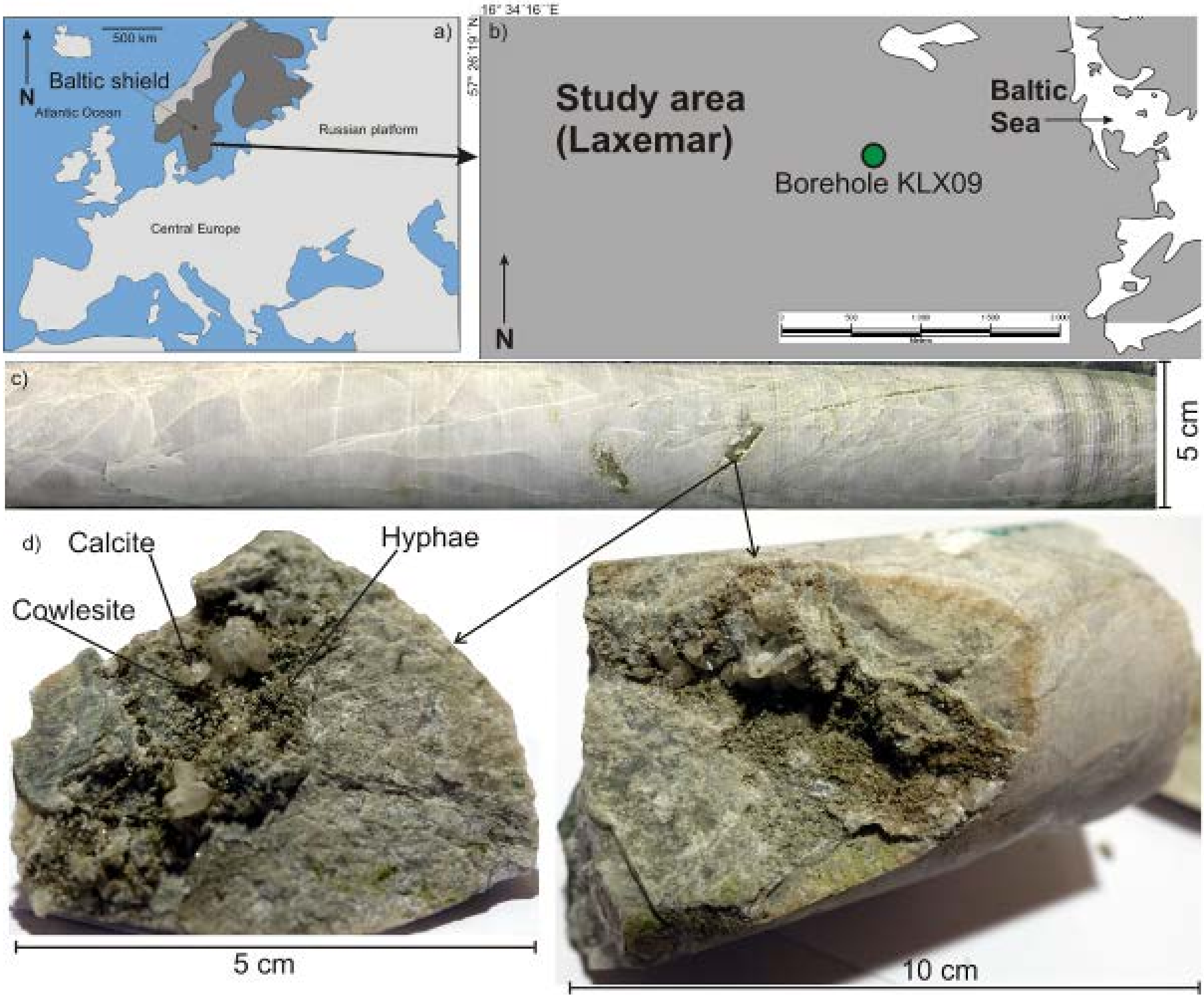

Supplementary Figure 1. (**a**) Map of Europe with study area in the Baltic Shield indicated. (**b**) Study area (Laxemar, Sweden), with sampled borehole indicated. (**c**) Photograph of drill core sample from 740 m depth. The sample consists of a quartz vein with a couple of porous sealed fractures (partly open fractures). (**d**) Detailed photographs of the euhedral minerals on the fracture surface of one of the fractures. Calcite, cowlesite and fungi hyphae are indicated by arrows.

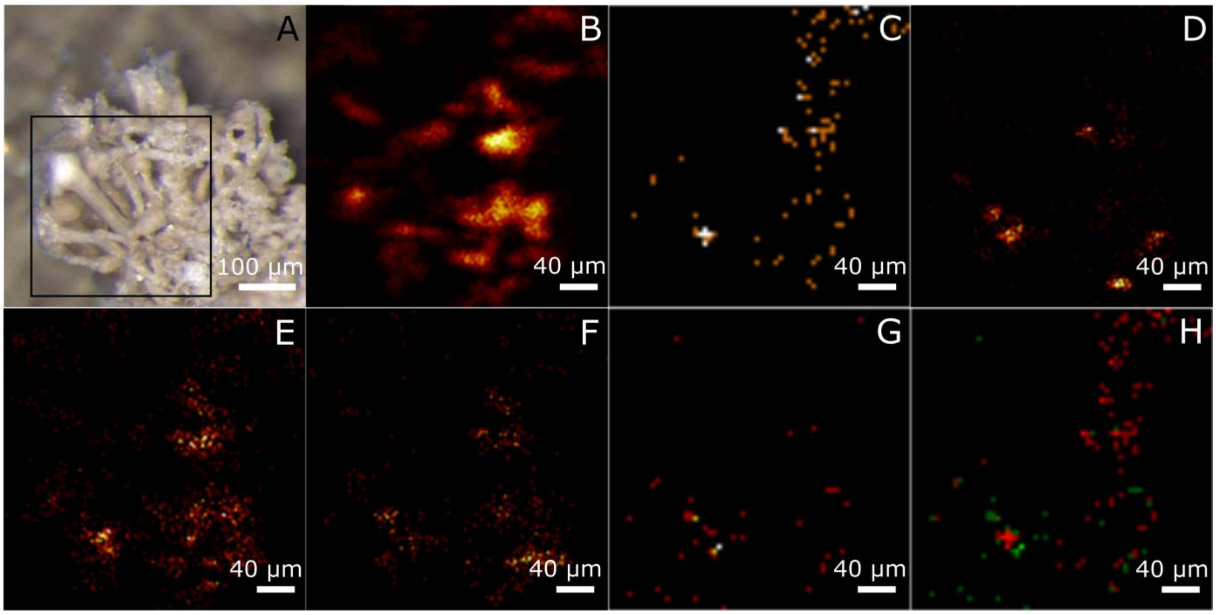

Supplementary Figure 2. ToF-SIMS analysis of area with hyphae. The different spatial distrubtion of the organic and inorganic ToF-SIMS signal indicates that the hyphae are partly mineralized. **A**) Micrograph of hyphae. Square indicate area of ToF-SIMS analysis in **B-G**. ToF-SIMS ion images of **B**) SiO_2 (m/z 59.96), **C**) sulfur (m/z 31.97), **D**) SO_4H (m/z 96.96), **E**) PO_2 (m/z 63.97), **F**) $C_3H_3O_2$ (m/z 71.02), **G**) fatty acids (added m/z 255.23 and 281.25), and **H**) Composite ion image of sulfur (C, red) and fatty acids (G, green).

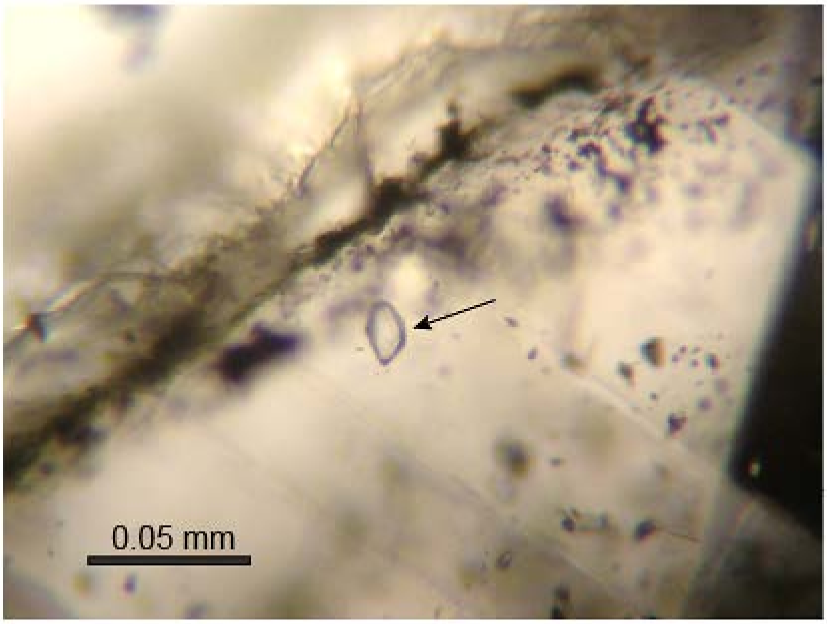

Supplementary Figure 3. Single phase (liquid) primary fluid inclusion in calcite.

Supplementary Table 1. Organic compounds detected using GC/MS.

m/z	Retention time	MW	Formula	Name	Characteristic fragments
156	8.46	156	$C_{12}H_{12}$	1,7-dimethylnapthalene	156; 141; 115
	8.51	156	$C_{12}H_{12}$	2,6-dimethylnaphthalene	156; 141; 115
168	8.96	168	$C_{13}H_{12}$	Diphenylmethane	168; 167; 165; 152; 139; 91
	10.14	168	$C_{13}H_{12}$	?-Methylbiphenyl	168; 167; 165; 153; 111 (high)
	10.31	168	$C_{13}H_{12}$	2-Methylbiphenyl	168; 167; 165; 153; 111, 83 (higher)
	11.02	182	$C_{14}H_{14}$	2,2-Dimethylbiphenyl	182; 167; 152; 89
	11.27	182	$C_{14}H_{14}$	2,3-Dimethylbiphenyl	
	13.42	198	$C_{15}H_{18}$	naphthalene? 1,6-dimethyl- 4-(1-methylethyl)/cadalene	
	13.46	198	$C_{15}H_{18}$	cadalene?	198; 183 (base peak) 168; 165; 155; 141; 128
m/z 196	13.01	196	$C_{15}H_{16}$	1,1,'-methylenebis(4-methyl) benzene	196; 181; 165; 104
	13.33	196	$C_{15}H_{16}$		
	13.62	196	$C_{15}H_{16}$		
	13.84	196	$C_{15}H_{16}$		
155; 170	10.59	170	$C_{13}H_{14}$	2-(1-methylethyl)-naphthalene	170; 155; 153; 128; 115
182					
64	19.58	256	S_8	Sulfur	256; 224; 192; 160; 128; 96; 64
	15.88	242	$C_{15}H_{30}O_2$	Tetradecanoic methyl ester (C_{14}-ME)	242; 199; 143; 87; 74
	19.89	270	$C_{17}H_{34}O_2$	Hexadecanoic acid methyl ester (C_{16}-ME)	270; 227; 199; 143; 87; 74
	23.57	298	$C_{19}H_{38}O_2$	Octadecanoic methyl ester (C_{18}-ME)	298; 255; 199; 143; 87; 74

Supplementary Table 2. Pyrite $\delta^{34}S$ values from borehole KLX09:740 m depth, coordinates (N: 57° 25′ 43,47″; E:16° 37′ 1,80″)

$\delta^{34}S$ (‰, V-CDT)	± ‰	Crystal
24.4	0.09	1
21.5	0.09	1
25.0	0.37	1
-22.6	0.19	1
23.0	0.09	2
23.0	0.09	2
20.3	0.12	2
-25.7	0.10	2
24.0	0.09	3
16.7	0.09	3
-27.8	0.08	3
-49.0	0.10	3
24.1	0.09	4
25.4	0.09	4
21.3	0.09	4
0.3	0.28	4
23.1	0.09	5
22.0	0.10	5
17.7	0.09	5
-14.5	0.21	5
26.3	0.10	6
24.6	0.09	6
19.4	0.25	6
-20.4	0.10	6
25.0	0.11	7
24.9	0.09	7
24.2	0.09	7
19.4	0.09	7
23.5	0.09	8
23.2	0.09	8
29.0	0.09	8
-37.3	0.10	8
23.2	0.09	9
21.8	0.10	9
-7.7	0.38	9
-53.3	0.13	9
21.1	0.09	10
24.0	0.09	10
30.9	0.13	10
-17.3	0.09	10
30.6	0.20	11
23.5	0.10	11

-24.4	0.08	11
-3.6	0.30	11
-21.0	0.08	12
-17.8	0.20	12
26.9	0.15	13
23.3	0.09	14
23.2	0.10	14
17.4	0.20	14
-36.6	0.11	14
12.3	0.12	15
18.8	0.09	15
-4.4	0.35	15
-33.7	0.13	15

S-isotopes were normalised using the Ruttan pyrite (+1.408‰ V-CDT[1]).

Supplementary Table 3. Calcite δ^{13}C and δ^{18}O values from borehole KLX09: 740 m depth, coordinates (N: 57° 25′ 43,47″; E:16° 37′ 1,80″)

δ^{13}C (‰, V-PDB)	± ‰	δ^{18}O (‰, V-PDB)	± ‰	Crystal
-29.2	0.53	-9.65	0.16	1
-29.5	0.52	-8.60	0.17	1
-28.1	0.52	-8.12	0.17	1
-34.1	0.51	-8.39	0.18	1
-35.5	0.51	-7.97	0.17	1
-17.0	0.53	-13.2	0.17	1
0.0	0.55	n.a.		2
-27.5	0.54	-4.36	0.18	2
-17.4	0.53	-14.1	0.17	2
-27.0	0.53	n.a.		3
-27.8	0.35	-6.29	0.21	4
-42.7	0.35	-7.25	0.20	4
-16.6	0.37	-12.4	0.20	4
-17.3	0.36	-12.4	0.21	4
-27.5	0.38	-7.76	0.21	5
-27.5	0.36	-10.4	0.22	5
-18.6	0.35	-12.8	0.20	5
-5.42	0.39	-19.5	0.19	6
-26.7	0.35	-6.41	0.19	6
-28.3	0.35	-7.61	0.20	6
-16.6	0.35	-11.9	0.19	6
-30.8	0.37	-15.1	0.21	7
-39.8	0.35	-5.59	0.21	7
-16.5	0.36	-11.6	0.20	7
n.a.		-14.5	0.20	7
-29.2	0.35	-6.56	0.21	8
-37.1	0.35	-6.75	0.21	8
-23.5	0.41	-13.5	0.22	8
-17.6	0.36	-11.2	0.19	9
-16.4	0.36	-12.3	0.20	9
-16.5	0.36	-11.6	0.21	9
-20.0	0.36	-13.9	0.22	9
-22.6	0.35	-13.7	0.20	9
-19.4	0.45	-11.1	0.20	10
-15.7	0.36	-12.6	0.21	10
-20.9	0.36	-15.4	0.19	10
-4.81	0.37	-20.9	0.21	11
-29.9	0.36	-7.91	0.19	11
-27.4	0.36	-7.51	0.21	11
-31.6	0.35	-7.06	0.21	11
-31.3	0.36	-7.32	0.23	11
-17.0	0.36	-6.96	0.20	11

-25.7	0.47		-6.69	0.38	13
-35.1	0.52		-7.48	0.35	13
-26.7	0.49	n.a.			13
-26.5	0.48		-7.72	0.34	13
-19.9	0.48	n.a.			13
-22.2	0.50		-13.1	0.38	13

Isotope data from calcite were normalised using calcite standard S0161 which comes from a granulite facies marble in the Adirondack Mountains, kindly provided by R.A. Stern (Univ. of Alberta). The values used for IMF correction were determined by conventional stable isotope mass spectrometry at Stockholm University on ten separate pieces, yielding $\delta^{13}C$ (PBB) = -0.22 ± 0.11 ‰V-PDB (1 std. dev.) and $\delta^{18}O$ = -5.62 ± 0.11 ‰ V-PDB (1 std. dev.).

Supplementary references

1. Cabral, R. A. et al. Anomalous sulphur isotopes in plume lavas reveal deep mantle storage of Archaean crust. *Nature* **496**, 490-493 (2013).
